# Supplementary material for: EEG microstate transition cost correlates with task demands
Source: PLoS Comput Biol. 2024 Oct 10;20(10):e1012521. doi: 10.1371/journal.pcbi.1012521 (PMC11495555; doi:10.1371/journal.pcbi.1012521)

S1 Fig. Selection of the best number of microstates using the cross-validation criterion. (*left*) Fraction of total variance (GEV) explained by the microstates. (*center*) Residual noise. (*right*) Cross-validation (CV) as a function of the number of microstates (N states).

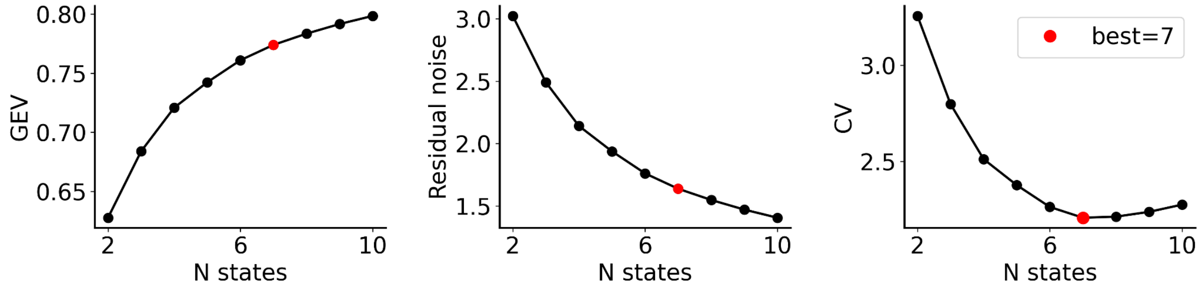

Supplement: S1 Fig — (left) Fraction of total variance (GEV) explained by the microstates. (center) Residual noise. (right) Cross-validation (CV) as a function of the number of microstates (N states). (PDF) [file pcbi.1012521.s001.pdf]
