# Supplementary material for: EEG microstate transition cost correlates with task demands
Source: PLoS Comput Biol. 2024 Oct 10;20(10):e1012521. doi: 10.1371/journal.pcbi.1012521 (PMC11495555; doi:10.1371/journal.pcbi.1012521)

S2 Fig. Behavioural results of the spatial Stroop task.

Distribution of reaction times (RT) for the 44 participants during each task condition.

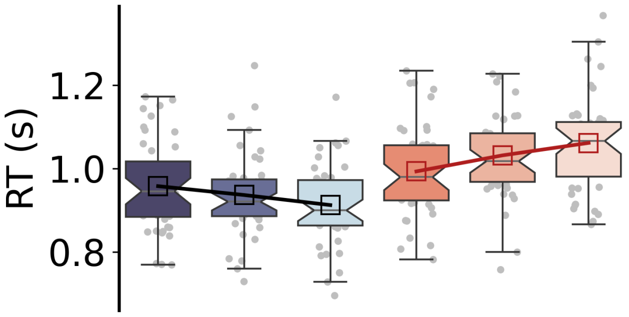

Supplement: S2 Fig — Distribution of response times (RT) for the 44 participants during each task condition. (PDF) [file pcbi.1012521.s002.pdf]
