# Supplementary material for: EEG microstate transition cost correlates with task demands
Source: PLoS Comput Biol. 2024 Oct 10;20(10):e1012521. doi: 10.1371/journal.pcbi.1012521 (PMC11495555; doi:10.1371/journal.pcbi.1012521)

S3 Fig. Global modulation of microstate distributions during the spatial Stroop task.
Distribution of Kullback-Leibler divergence (D_{KL}) between the task (π_{task}) and resting (π_{rest}) for the 44 participants.

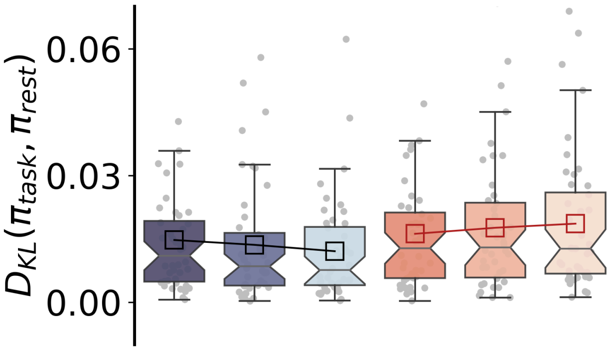

Supplement: S3 Fig — Distribution of Kullback-Leibler divergence (DKL) between the task (πtask) and resting (πrest) for the 44 participants. (PDF) [file pcbi.1012521.s003.pdf]
