# Supplementary material for: EEG microstate transition cost correlates with task demands
Source: PLoS Comput Biol. 2024 Oct 10;20(10):e1012521. doi: 10.1371/journal.pcbi.1012521 (PMC11495555; doi:10.1371/journal.pcbi.1012521)
Supplement: S1 Table — For each microstate, a linear mixed model was implemented to test whether the change in the probability distribution during task execution compared to the resting state is modulated by the stimulus congruency, the PC level, and their interaction. (PDF) [file pcbi.1012521.s004.pdf]

S1 Table. LMM results for microstate occurrences.

For each microstate, a linear mixed model was implemented to test whether the change in the probability distribution during task execution compared to the resting state is modulated by the stimulus congruency, the PC level, and their interaction.

State	Effect	*b*	*SE*	*t*	*df*	*p*	*d*	
A	Intercept	-0.018	0.004	-4.92	44.00	0.0000	-0.74	*
A	PC	0.000	0.001	0.15	62.03	0.8807	0.02	
A	Congruency	0.003	0.001	3.52	58.64	0.0008	0.46	*
A	Congruency:PC	0.005	0.002	2.51	101.65	0.0135	0.25	*
B	Intercept	-0.006	0.003	-2.12	44.00	0.0394	-0.32	
B	PC	-0.002	0.001	-1.68	49.60	0.0985	-0.24	
B	Congruency	0.005	0.001	5.14	48.49	0.0000	0.74	*
B	Congruency:PC	0.005	0.002	2.47	101.01	0.0153	0.25	*
C	Intercept	0.013	0.003	4.16	44.00	0.0001	0.63	*
C	PC	0.002	0.001	1.37	47.80	0.1770	0.20	
C	Congruency	-0.005	0.002	-3.07	44.56	0.0036	-0.46	*
C	Congruency:PC	-0.004	0.002	-1.63	105.23	0.1065	-0.16	
D	Intercept	0.010	0.003	3.61	44.00	0.0008	0.54	*
D	PC	0.000	0.001	-0.10	98.38	0.9219	-0.01	
D	Congruency	-0.002	0.001	-1.58	48.74	0.1196	-0.23	
D	Congruency:PC	-0.004	0.002	-1.64	67.94	0.1046	-0.20	
E	Intercept	-0.012	0.003	-3.96	44.00	0.0003	-0.60	*
E	PC	0.000	0.001	-0.15	44.11	0.8841	-0.02	
E	Congruency	0.003	0.001	3.13	44.19	0.0031	0.47	*
E	Congruency:PC	0.004	0.002	2.47	101.53	0.0151	0.25	*
F	Intercept	-0.007	0.004	-1.81	44.00	0.0767	-0.27	
F	PC	0.000	0.002	-0.24	44.12	0.8144	-0.04	
F	Congruency	0.005	0.001	4.65	44.16	0.0000	0.70	*
F	Congruency:PC	0.001	0.002	0.24	90.99	0.8140	0.02	
G	Intercept	0.021	0.002	10.09	44.00	0.0000	1.52	*
G	PC	0.001	0.002	0.39	44.25	0.6957	0.06	
G	Congruency	-0.010	0.001	-7.11	44.24	0.0000	-1.07	*
G	Congruency:PC	-0.006	0.003	-2.50	105.04	0.0141	-0.24	*

Notes: b, estimated coefficient; *SE*, standard error; *df*, degrees of freedom (estimated using the Satterthwaite method); The asterisks indicate the significant results after correction for multiple tests, applied using the false discovery rate approach.
