# Supplementary material for: EEG microstate transition cost correlates with task demands
Source: PLoS Comput Biol. 2024 Oct 10;20(10):e1012521. doi: 10.1371/journal.pcbi.1012521 (PMC11495555; doi:10.1371/journal.pcbi.1012521)
Supplement: S1 Text — Mathematical details on the modified k-means clustering algorithm. (PDF) [file pcbi.1012521.s005.pdf]

## S1 Text. Modified k-means clustering.

The modified k-means algorithm, introduced in [1], involves the traditional k-means clustering approach. However, it incorporates polarity-invariant topographical maps of the prototype microstates and considers the activation levels of these microstates, specifically their strength at each time point. In mathematical terms, each instantaneous activity map $x_t$ is assigned to the microstate index that minimizes the orthogonal squared Euclidean distance, $k_t = argmin_k d^2_{kt}$ , where $d^2_{kt} = x_t^T x_t - (x_t^T a_k)^2$. It turns out to be equivalent to maximizing the spatial correlation.

To define the best number of clusters, we employ the cross-validation criterion, which minimizes $CV = \sigma^2 (\frac{n_{ch}-1}{n_{ch}-K-1})^2$, where $\sigma^2 = \frac{\sum_t x_t^T x_t - (x_t^T a_t)^2}{T(n_{ch}-1)}$ is an estimator of the variance of the residual noise.

## References


1. Pascual-Marqui, R. D., Michel, C. M., & Lehmann, D. (1995). Segmentation of brain electrical activity into microstates: model estimation and validation. *IEEE Transactions on Biomedical Engineering*, *42*(7), 658-665.
