# Supplementary material for: EEG microstate transition cost correlates with task demands
Source: PLoS Comput Biol. 2024 Oct 10;20(10):e1012521. doi: 10.1371/journal.pcbi.1012521 (PMC11495555; doi:10.1371/journal.pcbi.1012521)
Supplement: Attachment — Submitted filename: Response_rewievers_Barzon_Plos.pdf [file pcbi.1012521.s006.pdf]

Dear Dr. Christoph Strauch,
Dear Dr. Lyle Graham,

Thank you for your email on February 22nd. We sincerely appreciate the valuable comments and constructive suggestions made by you and the reviewers for improving our article. Motivated by the overall positive feedback of the referees on the research question, the new version of the manuscript incorporates their suggestions and addresses their concerns.

In summary:

- We have performed the clustering analysis using the open-source “Pycrostates” toolbox (suggested by Reviewer #2) to align with standard pipelines in the microstate community and to increase the robustness and reproducibility of our results. The new results are nevertheless consistent with the ones contained in the previous version of the manuscript. All the new comprehensive preprocessed data and scripts for the analysis are reported in the updated GitHub repository.
- We have removed the terms “high”, “medium”, and “low”. Instead, we used the shorthand of proportion of congruency, PC. We have changed it on the manuscript and the figures (request by Reviewer #2).
- We have extended the description of the dataset (request by Reviewer #2).
- We have extended and clarified the mathematical description of our framework (request by Reviewer #2).
- We have extended the statistical analysis reporting, contained in the new method section. Furthermore, the report of the statistical results also includes the value of the statistics and the corresponding effect size (request by Reviewer #2).
- We have extended the discussion part about the neurobiological interpretation of the microstates (request by Reviewer #1).
- We have expanded the discussion section regarding the potential relationship between altered flexibility and control cost (request by Reviewer #3).

Point-by-point responses are attached below for your reference.

Sincerely,
Giacomo Barzon
Ettore Ambrosini
Antonino Vallesi
Samir Suweis

Reviewer #1:

Barzon et al. describe a new approach to develop a quantitative measure of "cognitive effort" using EEG microstates. The method is tested on a previously published dataset in which healthy participants perform a spatial Stroop task at three different levels of cognitive control demand.

They find:
- different microstate occurrences for different tasks
- transport cost depends on stimulus type, cognitive control level, and their interaction
- transport cost differences (Delta cost) correlates positively with reaction time (RT)

Strengths:
- The authors address a relevant question, i.e. how to estimate cognitive effort from neurophysiological data.
- They present a novel and innovative method. Transport theory has not been applied to EEG microstate data.
- The method is well described and reproducible.
- Microstate methodology follows standard procedures including an estimation of the optimum number of clusters (CV criterion).
- The experimental design has a tunable parameter PC (proportion of congruency) that gives a clear hypothesis about the actual cognitive demand.
- The results are well documented, quantified and statistically evaluated.
- The dataset has a good sample size, n=44.

We thank the reviewer for underscoring the strength of our work.

While the overall approach and the presented results promise a valuable and insightful publication, there is a number of open issues, listed below.

- Existing strategies to estimate cognitive load should be discussed in more detail. Previously used methods include spectral analyses but also EEG microstate analyses, in particular Jia et al., https://doi.org/10.1038/s41598-021-03577-1. To validate their new method, the authors should compare their results with at least one previously published approach (e.g. entropy rate, Hurst exponent).

We thank the reviewer for the suggestion. The above-proposed methods, however, are aimed at computing cognitive load during a single condition. Differently, our method does not estimate cognitive load during a single condition (i.e., the energy demand for “staying” in one condition), but the cost for transitioning between different conditions (i.e., the energy demand for “moving” between conditions).

Nevertheless, we computed the entropy rate as suggested by the reviewer using the “Pycrostates” package.

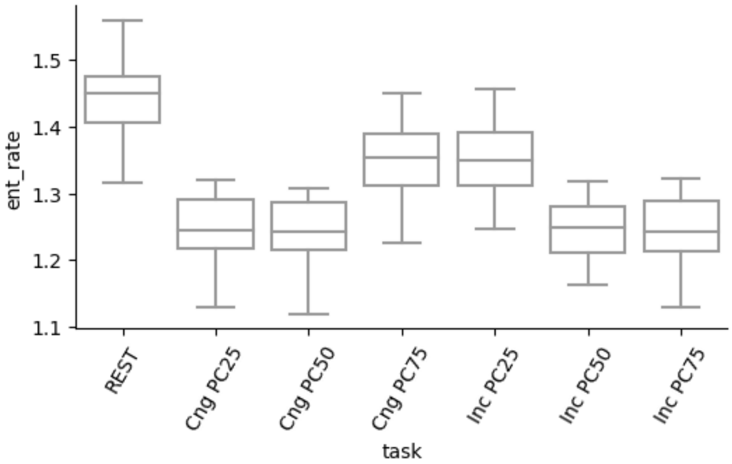

Figure R1: The boxplots show the distribution of entropy rate at rest and during the task conditions, defined both by the stimulus congruency and the PC level.

As shown in Figure R1, we found a higher entropy rate during rest and in conditions requiring less cognitive control. These results are consistent with the literature ("Escrichs, A., Sanjuán, A., Atasoy, S., López-González, A., Garrido, C., Càmara, E., & Deco, G. (2019). *Characterizing the dynamical complexity underlying meditation.* Frontiers in systems neuroscience, 13, 27.", "Saenger, V. M., Ponce-Alvarez, A., Adhikari, M., Hagmann, P., Deco, G., & Corbetta, M. (2018). *Linking entropy at rest with the underlying structural connectivity in the healthy and lesioned brain.* Cerebral Cortex, 28(8), 2948-2958.", "Capouskova, K., Kringelbach, M. L., & Deco, G. (2022). *Modes of cognition: Evidence from metastable brain dynamics.* NeuroImage, 260, 119489.") This may be related to the fact that "*... in rest, the brain's regime is orchestrated by increased metastability, meaning that the system's dynamics are more unpredictable or random and span a wider dynamical regime than in cognitive tasks.*" Moreover, "*... higher entropy might appear to be disadvantageous with increased randomness, but in a condition when no instant reaction is required, such a state is desirable, as higher entropy allows for more easily learning a new task with lower specialized performance ... Rest can, therefore, be viewed as a preparatory off-line state that provides the grounds for more efficient performance during a specific on-line task.*"

A microstate-based measure that could potentially estimate cognitive load is entropy production (Lynn, C. W., Cornblath, E. J., Papadopoulos, L., Bertolero, M. A., & Bassett, D. S. (2021). *Broken detailed balance and entropy production in the human brain.* Proceedings of the National Academy of Sciences, 118(47), e2109889118.) As we briefly discussed, this measure is related to the asymmetry of transition probabilities, which we have found to be true even at rest. However, to our knowledge, this measure has not yet been explored in the EEG field, so we leave this for future work.

- The proposed transition cost measure has an interesting interpretation in terms of brain state switches. Whether these transition dynamics (Q_ij) are really relevant could be tested by comparing with a simpler approach that looks at the shape of the microstate distribution only. For example, does the entropy of the microstate distribution alone (in each condition) perform worse than the proposed measure that takes into account Q_ij?

We thank the reviewer again for the suggestion. However, we would like to emphasize that measures that consider the microstate distribution, such as the Kullback-Leibler divergence (as previously explored in “Capouskova, K., Kringelbach, M. L., & Deco, G. (2022). Modes of cognition: Evidence from metastable brain dynamics. NeuroImage, 260, 119489.”), do not have a clear interpretation in terms of transition cost because they do not account for dynamics. Furthermore, a condition with a large KL divergence may, in principle, have a small transition cost if changes in the microstate distribution align with more favored (i.e., more probable) transitions at rest.

Nevertheless, we also compared the microstate distribution during rest and various tasks by computing the Kullback-Leibler divergence between the microstate distribution.

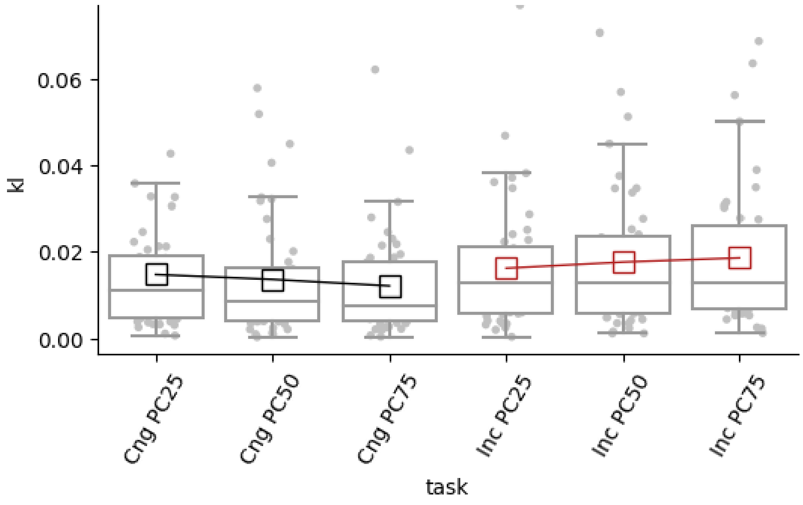

Figure R2: The boxplots show the distribution of the Kullback-Leibler divergence between the distribution of microstates at rest and during the task conditions, defined both by the stimulus congruency and the PC level.

We found that the KL divergence aligns with the expected levels of cognitive demand (see Figure R2). This is consistent with the modulation of microstate distribution across different tasks, as reported in Fig. 2. We reported such analysis in the new Figure S3, and we added this considerations to the discussion section.

- p.4, Fig. 1: "found in the literature [Michel et al. 2018], and we labeled them accordingly (from A to E)". Microstate class E (ms-E) shown in Fig. 1b is very different from ms-E in Michel et al., NeuroImage, 2018, or in Custo et al., 2017. The authors should discuss this and consider using a different label for this map.

Upon re-examining the microstate analysis (using the “Pycrostates” package - as detailed below), we now identified K=7 clusters, which aligns with the maps consistently found in the literature (see Custo et al., 2017). Therefore, we have decided to retain the naming convention A to G for consistency.

- p.8, microstate backfitting: "The EEG map at each time point was labeled according to the map...". Does this imply that maps were back-fitted at each time point without any temporal smoothing (interpolation, parametric smoothing, minimum duration etc.)? If yes, this should be made explicit as smoothing is very common in the microstate community

Thanks to the reviewer's comment, we recognized that our custom pipeline differed from common practices in the literature. Specifically, we applied a smoothing step (Gaussian kernel of 5 timesteps) prior to the clustering procedure. Additionally, we conducted clustering on the concatenated GFP peaks from each subject.

In order to align with standard practices in the scientific community, we opted to use the open-source package "Pycrostates" for clustering. As described in the revised manuscript, we implemented a two-level clustering approach: first, at the single-subject level to estimate individual maps, and then at the group level to derive group templates. We refrained from applying any smoothing to preserve the dynamics of the microstates.

The revised version of the manuscript contains the updated results obtained with the new clustering analysis.

- The authors use the term "occurrence" throughout the manuscript. While it becomes clear what they are referring to, I would suggest to switch to "coverage" or "distribution" as occurrence is a commonly used but different microstate parameter that refers to the frequency with which a given microstate class occurs and therefore has units of frequency (1/s). This could easily confuse readers used to the 'standard' terminology.

We thank again the reviewer for pointing out this inaccuracy. We have now used the term “distribution” throughout the revised manuscript.

- The authors identify ms-A as indicative of the resting condition (e.g. Fig. 2). This is very unusual, ms-A is commonly associated with task performance and rest is often associated with ms-C (ms-D), probably representing posterior dominant resting state rhythms (alpha). This must be discussed and further assessed. Is this attributable to the relatively large number of clusters (K=9)? Does the finding persist in the case K=4?

We thank the reviewer for pointing out this issue. As observed from the analysis with K=4 reported below (see Figure R3), also in this case microstate A seems consistently more prominent during rest compared to task execution. This, however, does not imply that its expression is not significantly higher at rest compared to the chance level (see “Microstate reconfiguration during task” section).

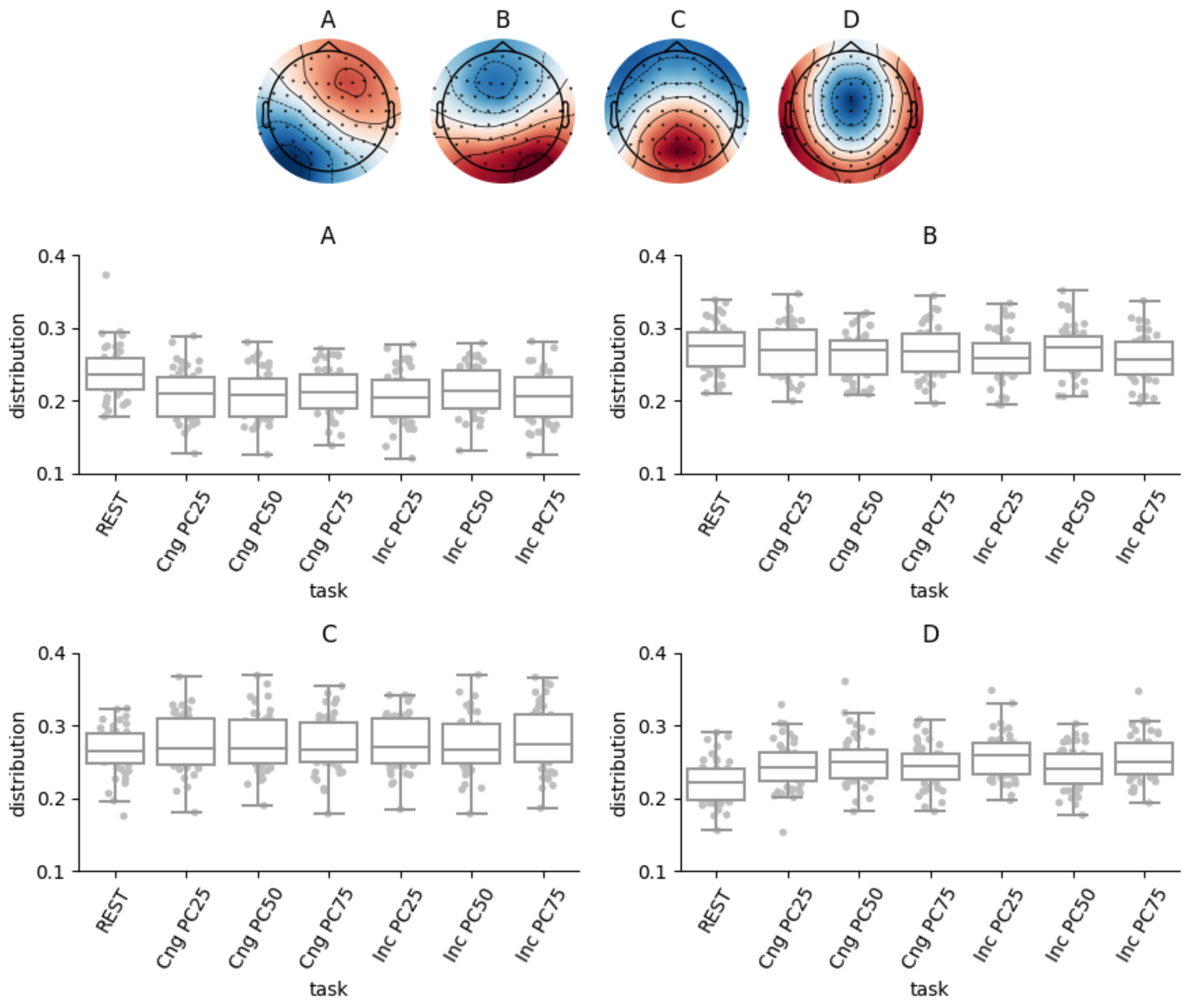

Figure R3: The boxplots show the distribution of the microstates obtained from the clustering by fixing the number of centroids K=4. The corresponding group maps are shown at the top.

While this finding may appear contradictory to existing literature, we acknowledge that without source analysis, understanding the specific neural activity associated with each microstate is challenging, as discussed further in the manuscript.

We note that the updated analysis microstates C was suppressed during tasks (see Fig. R3), which is coherent with the existing literature [Custo et al., 2017; Michel et al, 2018] as suggested by the referee. Instead, we believe that source reconstruction analysis is the only way to access the functional interpretation of microstate A.

- Please provide more quantitative information about the analysed trials. Apparently, microstate distributions were obtained for each presented stimulus. How long were these trials on average and how many microstates were found during one stimulus/response trial? From Tafuro et al., I understand that participants had 750 ms to initiate movement, 2000 ms to complete, and 1500 ms blank post-stimulus. Let's say an average trial lasts about 2000 ms (assuming that participants complete in much less than the given 2000 ms) and the average microstate duration is 100 ms. This gives only 20 microstates per trial and as little as 2 samples/histogram bin for K=9 microstates. Please provide the actual number of samples that went into the estimated microstate distributions. Within a block (high, medium,

low), were the microstate distributions for all C- (or I-) trials pooled? Were microstates at trial boundaries excluded?

We apologize for the lack of clarity. As noted by the reviewer, each participant had 2000 milliseconds to complete the task. Considering that each microstate lasts an average of approximately 20 milliseconds (as shown in Figure R4 below), each block comprises approximately 100 microstates.

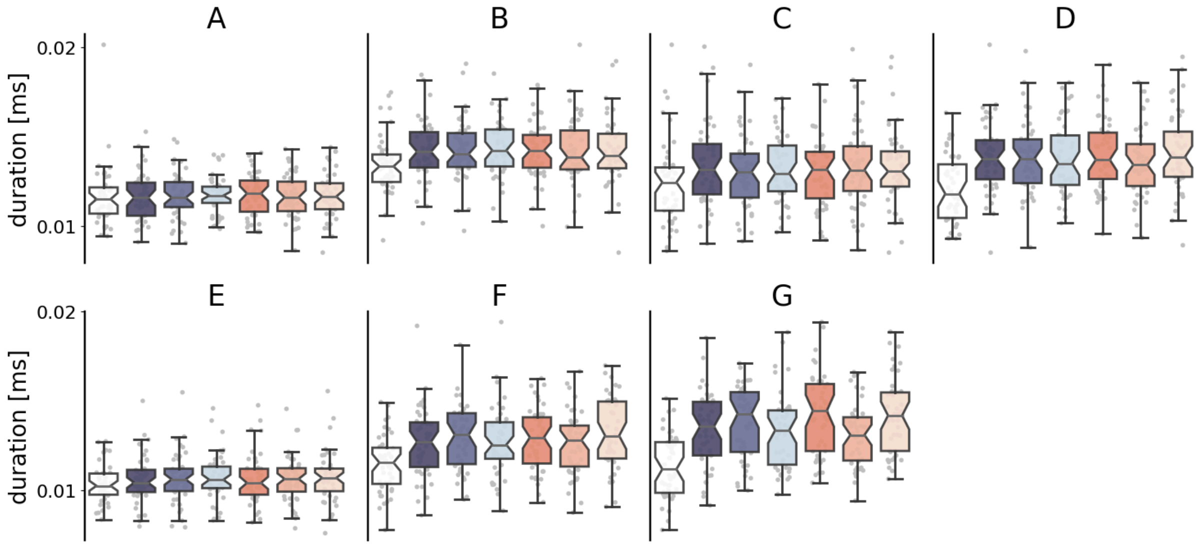

Figure R4: The boxplots show the distribution of the durations of each microstate during resting and the different task conditions.

Furthermore, because the order of C-I trials within each block was randomized, we combined all trials related to each condition (Cong-PC25, Cong-PC50, Cong-PC75, Incong-PC25, Incong-PC50, Incong-PC75), disregarding the inter-trial intervals, and excluded microstates occurring at trial boundaries. Thus, the microstate distribution was computed based on approximately (100 x number of trials) microstates for each condition (i.e., approximately 7200 microstates for the PC50 and PC25 conditions and 21600 for PC75 conditions).

To be consistent with the other conditions, we have also chunked the resting state into 2000 ms blocks, again discarding the microstates at the boundaries.

These details have now been included in the "EEG microstate-based analysis" section of the methods.

- p.4: "In essence, this cost was calculated as the disparity between the spontaneous microstate dynamics during the resting phase and the bridge".
This is where my most serious doubt lies. While the approach is clear and meaningful in general, this does not seem to reflect the experimental reality of the dataset. From Fig. 1a, I understand that the resting phase occupied the first 4 minutes, followed by the task phase that switches between high/medium/low blocks and C/I stimuli within each block. This means that the actual brain state transitions occurred between different task conditions (e.g. C-M and I-M, or between high and medium), but not between rest and task. Is the bridge calculation rest-task meaningful in this context? Does the approach still work when you

calculate transition costs between the different tasks as they occurred during the actual experiment? Do the authors assume that subjects return to the resting state between consecutive stimuli? If yes, can this be justified from the literature?

As correctly pointed out by the reviewer, the transition cost was calculated between the microstate distributions during the various tasks and the initial resting. Computing the actual cost would require computing statistics not over the (static) distribution of microstates but on actual trajectories. This, unfortunately, is infeasible, since it would require an incredibly large amount of data (as also discussed in Kawakita, Genji, et al. "Quantifying brain state transition cost via Schrödinger bridge." Network Neuroscience 6.1 (2022)). Nevertheless, the cost can still be estimated by inferring the most probable trajectory linking these distributions using the Schrodinger bridge approach.

This approach is consistent with the current literature on control theoretical approaches for estimating the transition cost, which is computed between an initial resting phase and the various task conditions (see e.g., “Tang, E., & Bassett, D. S. (2018). Colloquium: Control of dynamics in brain networks. Reviews of modern physics”; “Ceballos, et al (2024). The control costs of human brain dynamics. bioRxiv”; “Kamiya, et al. (2023). Optimal control costs of brain state transitions in linear stochastic systems. Journal of Neuroscience”; “Kawakita, G., et al. Quantifying brain state transition cost via Schrödinger bridge. Network Neuroscience, 2022”).

In addition, we would like to highlight that each stimulus was followed by a resting phase of 1500 ms, while “...*breaks were included at the end of each block, so that participants could rest and decide when to continue with the following block.”* [Tafuro et al, 2020].

- p.5 "our results hint at a macroscopic entropy production..." - consider moving this to the Discussion section.

We agree with the reviewer's suggestion and have moved the statement regarding macroscopic entropy production to the discussion section.

- "stimulus type" is only used twice on page 6. As it is an important part of the results, please introduce the term in the Methods section.

We have made its usage clearer throughout the manuscript.

- Fig. 4a: The bracket on the right (single asterisk) seems to compare C-low and I-low. Is this a mistake and should the bracket indicate the comparison I-high and I-low instead? Please clarify.

We thank the reviewer for the comment. In the previous version, we performed post-hoc to also compare congruent and incongruent trials for each value of PC, since this is an estimate of the Stroop effect, which is the standard measure of the interference resolution ability in the cognitive control literature. However, in the analyses we reported in the revised version of the manuscript, we modeled PC as a continuous predictor, so to avoid the need of post-hoc tests (as suggested by Reviewer #2). In the current version, we have added a

section with a detailed explanation of the statistical analysis, which also reported the estimation of the Stroop effect.

- Fig. 4b: The correlation is not very clear from the plot. "revealed a significant positive correlation within this distribution" - does this refer to the whole data cloud shown in Fig. 4B? Can the authors please add a line indicating the correlation?

We apologize again for the lack of clarity. We have now updated Fig. 4b by reporting the random effect of the linear mixed model between the participants' Stroop effects in transition costs (i.e., the difference in transition costs between incongruent and congruent trials: Δ Cost) as a function of their Stroop effects in response times (Δ RT).

- The Discussion section is extremely short regarding the neurobiological interpretation and the potential practical value of the results.
Points that should be addressed include:
a) Why does ms-A appear as an indicator of the resting state (and not ms-C, as reported in the literature)?

We thank again the reviewer for pointing out this limitation. In the revised version, we have extended the discussion about the possible interpretation of the microstates. As already noted, in the updated analysis microstates C was suppressed during tasks, which is coherent with the existing literature as suggested by the referee. Instead, we believe that source reconstruction analysis is the only way to access the functional interpretation of microstate A.

b) Fig. 4a, why are transition cost differences more pronounced for the easier stimulus type C (congruent)? Shouldn't the transition cost differences be larger for the more demanding incongruent (I) type?

The revised analyses suggest that, for incongruent stimuli, the transition costs are higher, on average, and increase in a PC-dependent way.

c) Fig. S4 shows that RT is large for I-low and C-high. Doesn't this mean that RT is an inconsistent measure of cognitive demand?

That pattern (see Fig. S2 in the revised version) is consistent with the standard findings in the adaptive control literature of an increase of the RT Stroop effect in conditions with higher PC levels (that is, when cognitive control demands are lower; see for instance the papers from our lab that are cited in the manuscript). The RT for incongruent stimuli are indeed commonly found to be higher in conditions with higher PC levels (due, again, to the lower level of cognitive control); by contrast, the RT for congruent stimuli are commonly found to increase in conditions with lower PC levels, a finding that is called "congruency cost" (see also "Gonthier, C., Braver, T. S., & Bugg, J. M. (2016). Dissociating proactive and reactive control in the Stroop task. Memory & Cognition, 44, 778-788."), because the associated higher levels of cognitive control imply to bias participants attention away from the irrelevant stimulus feature.

d) Given the variance, is the effect size observed for incongruent stimuli shown in Fig. 4a relevant for practical use at all? There seems to be (almost) no change in transition cost although the PC varies between 75% and 25%.

The effect size for the PC effect in incongruent stimuli is d = 0.33, corresponding to a small-medium effect and, therefore, we think that it is relevant for practical use. It is important here to note that the variability reported in the boxplots is the between-participants variability in each condition, which we reported for the sake of completeness, but it is not relevant to the computation of the statistical tests and the corresponding effect sizes, which instead rely on the variability of the differences across conditions (because we used a within-subjects design). In other words, even if the between-subjects variability is large in each condition, the size of a within-subjects experimental effect can still be large if the corresponding between-condition difference has a low between-participant variability (i.e., if the experimental effect is stable across participants).

e) Although statistically significant, is the correlation shown in Fig. 4b of any practical value? How large is the correlation coefficient? I hypothesize that the correlation is significant only due to the congruent trials. The cost for incongruent tasks looks constant in Fig. 4a, so the difference congruent-incongruent follows the congruent data. Why did the authors assess the difference values, are they of any biological relevance (are they used in the literature?).

In the revised analyses, the relationship between transition costs and performance has a medium effect size of d = 0.45. Moreover, we chose to test this effect by using the Stroop effect, that is, the difference between incongruent and congruent trials, as it is the golden-standard measure of performance in the Stroop task.

Overall, as the authors promise "fresh perspectives for physiologically describing cognitive effort", the results require a more critical evaluation. There are some positive correlations for congruent trials but the discrepancy between congruent and incongruent tasks makes the results much less convincing (unless explained further).

Minor points:

- The greek letter pi occurs as both upper and lower case letters throughout the manuscript and Figures, please use one form only.

We have standardized the use of the Greek letter pi to the same lower-case form throughout the manuscript and figures.

- The term in Fig. 1c is "min D_KL", on p. 9 it's "min KL"

We also have standardized the use of D_KL throughout the manuscript to indicate the Kullback-Leibler divergence.

- Fig. S2: See above, "occurrence" can be confusing as most microstate papers define occurrence differently and a unit of 1/s is expected.

We switch to the term “distribution” as stated above.

We are really grateful to the reviewer for the detailed and precise comments that we believe has enhanced the validity of our manuscript. We hope that in its current form our work may be considered for publication.

Reviewer #2:

This manuscript tries to link the construct of cognitive effort to the transition cost between multiple brain states. This research question is answered by using electrophysiological indexes for those quasi-stable brain states, i.e. micro-states, in the context of a cognitive control demanding task, a spatial Stroop task. The authors use the difference in microstates occurrence between a resting-state and the three different cognitive control level conditions of the task to show evidence for the link between cognitive effort, task conditions and ultimately behavior. In this paper, the proxy used for cognitive control is the transition cost between the different states that is estimated through a novel method for EEG.

At the general level, the paper presents an interesting approach, namely, how to assess cognitive effort through the estimation of transition costs among quasi-stable brain states.

We appreciate the reviewer's recognition of the novelty and significance of our work.

The paper, however, presents a strong lack of details in the methods but also the reported results. This lack impacts not only the replicability of the method to other contexts but also simply the understanding of most analysis in the paper. I come back to this issue among the several major points that I think need to be addressed. Parallel to this lack of methodological aspects, or maybe related, it looks like the statistical analysis chosen by the authors doesn't allow to tell whether the data supports the conclusion of the authors.

We apologize for the lack of clarity and detail in the methodological section of our paper. We have made significant efforts to improve our manuscript, addressing each concern raised by the reviewer. Below, we provide detailed explanations for each point.

Because of these problems my main comments on this manuscript are on the methodological side rather than on the research questions per se. In the following, I detail my main concerns for each section of the results.

On the microstates occurrences:
- I am quite surprised by the lack of variance among the state occurrences. Based on Fig S2 (which would better sit in the main text given its importance) it does look like every state has a probability of roughly 1/9 (and 9 happens to be the number of microstates). This uniform distribution of state occurrences doesn't seem to be true in other microstates paper (e.g. Milz et al. 2016) and, to me, questions whether the microstates extracted are really task related. I would suggest that the authors explore ways of assessing whether their clustering is robust, e.g. resampling in the context of microstates https://pycrostates.readthedocs.io/en/latest/generated/auto_tutorials/cluster/10_subject_level_with_resampling.html

Since the reviewer suggested to use the function contained in the "Pycrostates" toolbox, we recognized that the custom pipeline for the microstate clustering had some differences from our former custom pipeline. Specifically, we applied a smoothing step (Gaussian kernel of 5 timesteps) prior to the clustering procedure, which we found is not commonly done. Additionally, we conducted clustering on the concatenated GFP peaks from each subject, not doing the two-level analysis. In order to align with standard practices in the microstate

community and to increase the robustness and reproducibility of our results, we opted to use the open-source package "Pycrostates" for clustering. Thus, the revised version of the manuscript contains the updated results obtained with the new clustering analysis, which are nevertheless quite consistent with the previous ones.

In the revised version, we proved that the microstate distribution (at rest) significantly differs from the uniform distribution. This “lack of variance” may be attributed to the larger number of microstates compared to Milz et al., 2016. Indeed, if we perform the clustering using (the non-optimal) K=4, we found that the variance among microstate distribution is increased (see Figure R5).

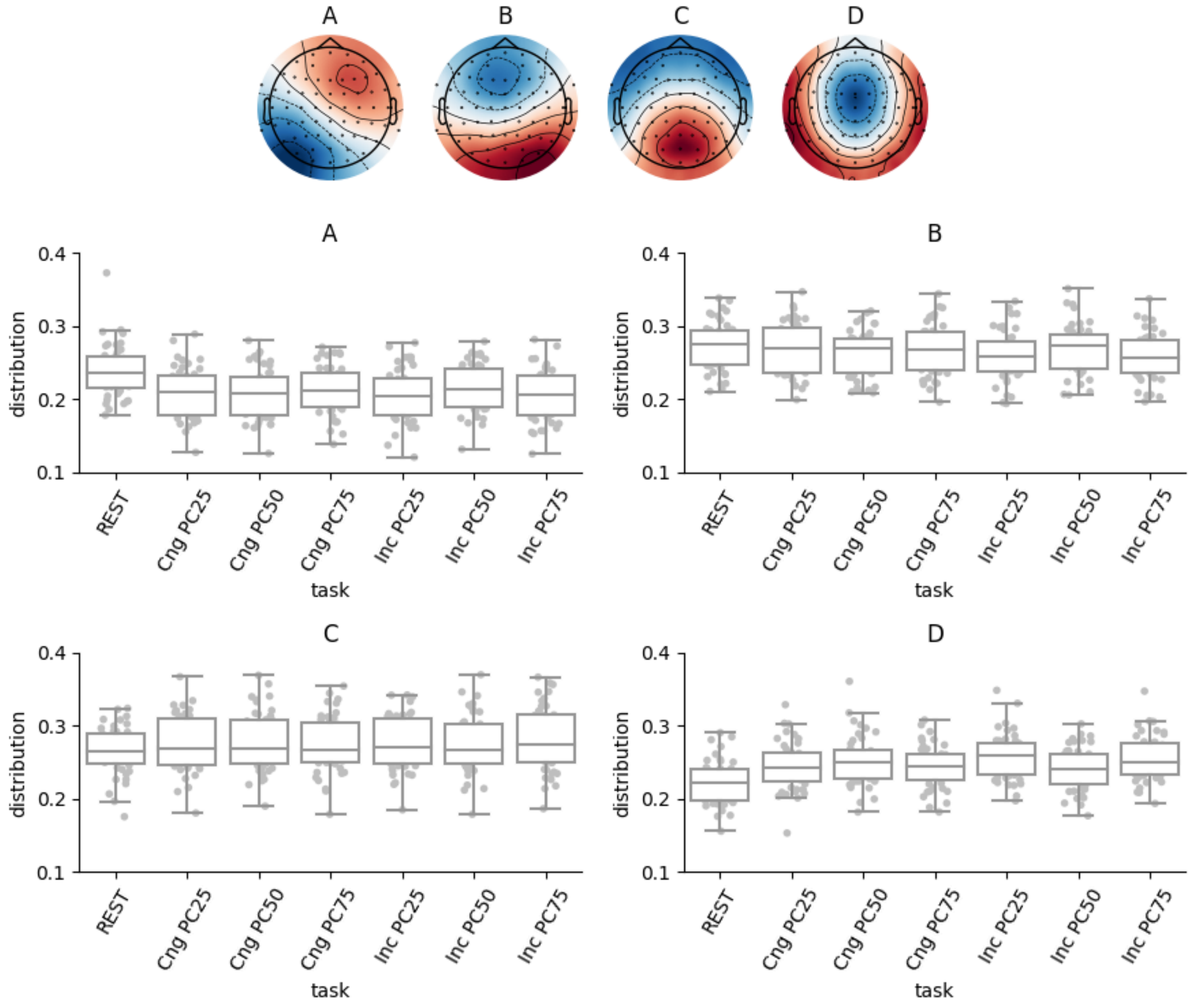

Figure R5: The boxplots show the distribution of the microstates obtained from the clustering by fixing the number of centroids K=4. The corresponding group maps are shown at the top.

We also confirmed whether the clusters are robust to resampling even at the individual level, as we can appreciate from Figure R6 below.

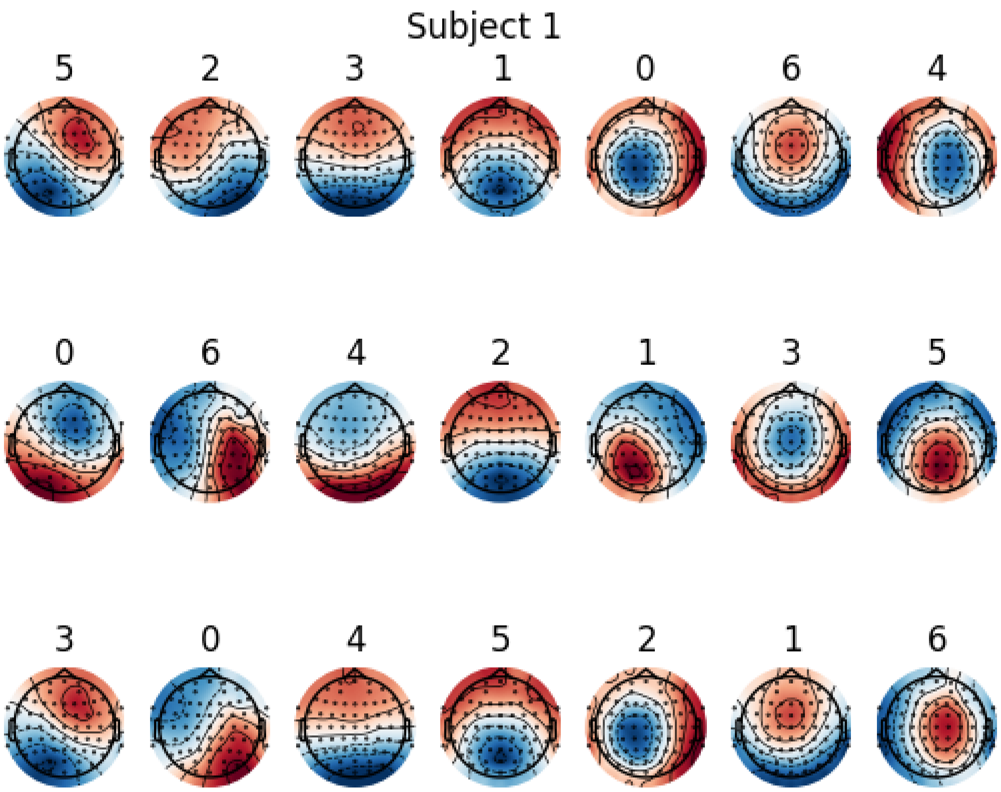

Figure R6: Examples of microstate maps from a single participant (subj. 1) obtained from the clustering with different resampling of GFP peaks.

Following the reviewer suggestion, we have also moved Figure S2 to the main text, which corresponds to the updated Figure 2.

- On the linear mixed model. First I am surprised that the author chose a linear model and not a generalized linear model as the normality assumption of the residuals implied by a linear model cannot be respected in a change of probability of occurrence. That is, maybe this assumption is reasonable if those changes in occurrences are close to 0.5 but this is clearly not the case (Fig. S2). More importantly, the model description (also in the supplementary material) is insufficient to understand and evaluate the statistical model. What contrast coding was applied to the factors (e.g. reference/intercept is compatible condition? how were the three categories of control level coded?), what random structure was chosen? Moreover the post-hoc test applied could be avoided by having a better modelization. In this case, the Bonferroni correction will increase type II errors which might hide interesting results from this analysis.

We thank the Reviewer for the comment, which allowed us to improve our manuscript. As suggested, in the revised version of the manuscript we better clarified our analyses (see "Statistical Analysis" section. We also improved our modelization to avoid applying post-hoc tests, as suggested. Finally, we performed residual analyses, "*which verified the assumptions of homoscedasticity and normality of the residuals and did not reveal relevant signs of stress in model fitting*" (see "Statistical Analysis" section).

Transportation cost matrix: I fail to understand the method. This might be due to my mathematical limitations and/or to an under-reporting of the method. In either case, this method being the core of the paper, it should be explained more thoroughly (e.g. what is P_{ij} in the equation on p. 9) and clearly (e.g. while I think I understand the parenthesis for Q_{ij}, I fail to see the link with the associated sentence).

We apologize for the lack of reporting on the method. We have now expanded the "Brain transition cost" section in the methods and also merged it with the additional descriptions that were left in Supplemental Text S2.

transition cost reflects task demand:
- I don't understand why the authors choose a two-way ANOVA. It seems to me that the measures in this case are also repeated across participants. Hence at the minimum a repeated measure ANOVA needs to be conducted or, for more coherence with the microstate occurrence analysis, a linear mixed model. Without this control it is impossible to support this analysis, even more so in the absence of any index usually reported along with the p-values (i.e. no F statistic or degrees of freedom).

We thank the Reviewer for the comment, which gave us the opportunity to improve our manuscript. As suggested, in the revised version of the manuscript we employed the same analytical approach for this analysis, conducting an LMM instead of the repeated measure ANOVA we used in the previous version of the manuscript (which we reported as a 2-way ANOVA). Now we also report the detailed statistics.

- For the link between reaction time and transition cost also the analysis is hard to understand because of the lack of reporting. The authors choose to compute correlations for each participant between RTs difference and cost. Is this between control levels? If so, I don't think a correlation is appropriate as 1) it is only for three points, 2) no guarantee exists on the linear link between RTs and cost difference across the three levels and 3) the second step analysis of the t-test ignores the uncertainty in the correlation. Assuming that point 2) is verified a better modelling approach would be to use again a linear mixed model and report the coefficient along the p-values. This would then resolve point 3) and, based on my understanding of what the authors did, be a proper test for the hypothesis laid out in this paragraph.

Again, following the Reviewer's suggestion, we conducted an LMM on Stroop effects, assuming a linear effect of PC on them, based on existing evidence and our previous findings, as reported in the "Statistical Analysis" section.

Minor:
- p.8 "To this aim, we recorded EEG in 44", implies that the data was recorded for this study

We apologize for the oversight. We have corrected the statement in the manuscript. We thank the reviewer for bringing it to our attention.

- p.5 last sentence of first paragraph, "Instead", poor wording?

We have revised the sentence for better clarity.

- The terms High, medium and low to describe 25, 50 and 75 % of congruent trials is a conflict in itself. It would be easier to read if the proportion of incongruent trials was reported instead

We have revised the terminology to avoid confusion. We have now reported the proportion of congrunet trials during each block instead of using terms like "high," "medium," and "low". These changes have been made both in the text and in the figures for better clarity.

- In p.2 I had difficulties following what exactly transition cost were defined as in relation to the research question, the Result section is however clearer. For the sake of readability, the authors may want to rewrite this section by explicitly relating to microstates in resting vs task.

We have revised the section to explicitly relate transition costs to microstates in resting versus task conditions for improved readability.

- p. 6 last paragraph, "subjective performance" to describe RT seems surprising as RT doesn't usually qualify as a subjective measure.

We apologize for the confusion. By "subjective performance," we meant the performance of each subject. We have now corrected this in the manuscript accordingly.

- p. 8 Dataset section insufficiently describes the dataset, how many electrodes, what reference was used, the manuscript should be self-standing for such aspects

We have now provided more detailed information about the dataset in the Dataset section.

Sincerely,
Gabriel Weindel

New references:
Milz, P., Faber, P. L., Lehmann, D., Koenig, T., Kochi, K., & Pascual-Marqui, R. D. (2016). The functional significance of EEG microstates—Associations with modalities of thinking. _Neuroimage_, _125_, 643-656.

We thank the referee for pointing us to this relevant piece of literature, that we have considered in the new version of the manuscript.

We are grateful for the thorough and detailed feedback provided by the reviewer, which has significantly improved the quality of our manuscript. We believe that our work, in its current form, can be considered for publication.

Reviewer #3:

This research paper focuses on the dynamics of the brain, describe as microstates as observed in EEG, and their role with respect to cognitive processes. In particular, the authors investigate how brain activity correlates with the level of cognitive exertion during tasks by employing EEG data. These studies apply the principles of optimal transport theory and the Schrödinger bridge problem to examine the changes and dynamics in the brain as it responds to varying cognitive challenges.

I think this paper is sound, and I do not have strong objections to it. In particular, I thought it was interesting to note that employing optimal transport theory aids in comprehending the brain's dynamics and the cognitive effort required during tasks by transforming the Schrödinger bridge problem into an entropy-regularised optimal transport issue. This method facilitates the adjustment of brain dynamics to accommodate cognitive needs by altering the distribution of EEG microstates, intensifying some while diminishing others. By identifying the most cost-efficient transportation plan, which incorporates an entropic regularisation component, researchers can study the variations in EEG microstates throughout tasks, identifying greater costs linked to cognitive shifts. The transportation cost matrix represents the cost of moving mass between supply (resting) and demand (task) locations, offering insights into the brain's strategies for handling and adapting to different cognitive demands. This approach presents an innovative method for evaluating cognitive effort through brain data, underlining the connection between patterns of neural activity, cognitive strain, and behavioural outcomes.

We sincerely thank the reviewer for the positive and constructive feedback on our paper. We are pleased to hear that you find our application of optimal transport theory to investigate the brain correlates of cognitive demands both interesting and effective.

At the end of the article, the whole scenario is clear, but at the level of the following sentence, I was confused. Hence, in my opinion, it would help to explain more clearly why it is possible to not use a mathematical modelling.

We apologize for the lack of clarity. We have now revised the paragraph.

It would be interesting to also have the same analysis in a pathological context to explore whether pathological conditions could influence the control cost, because has been observed that in pathological conditions we have a reduced flexibility in brain dynamics linked to different sequences of patterns.
For future research, can be a good idea to focus more on brain dynamics and study the evolution of these microstates across EEG data, implementing a mathematical model which, starting from a realistic framework activity, can also implement the Schrödinger bridge problem to reproduce a similar analysis. In particular, some papers share of the motivations with these paper, and have explored complexity and flexibilithy in pathology, such as: Cipriano, Lorenzo, et al. "Flexibility of brain dynamics is increased and predicts clinical impairment in Relapsing-Remitting but not in Secondary Progressive Multiple Sclerosis." medRxiv (2023): 2023-07; Polverino, Arianna, et al. "Flexibility of fast brain dynamics and disease severity in amyotrophic lateral sclerosis." Neurology 99.21 (2022): e2395-e2405; and Sorrentino, Pierpaolo, et al. "Flexible brain dynamics underpins complex behaviours as

observed in Parkinson’s disease." Scientific reports 11.1 (2021): 4051. In my opinion, this is relevant literature in this context.

We agree with the reviewer that extending our analysis to a pathological context is particularly intriguing. Exploring how pathological conditions might influence control costs, given the observed reduced flexibility in brain dynamics in such conditions, is an excellent direction for future research.

We also thank the reviewer for bringing to our attention the relevant literature on altered complexity and flexibility in pathology. We have now included these references in the discussion section.

In the abstract, I would suggest describing in a very concise way what is the Stroop task to give immediately the main idea of the work.

We appreciate the reviewer's suggestion. We have now included a concise description of the Stroop task in the abstract.

In the introduction, after the following sentence, you can add a reference:
<< At the macroscale, brain activity is characterized by spatially distributed groups of regions that exhibit temporally correlated activity and co-activate during behavioral tasks, thus acting as functional networks>>.

We have now included a reference after the sentence mentioned in the introduction.

The results and methods used are well described, clear and well explained. The statistical analysis done is sufficient.

All in all, I find this is a sound piece of scientific work, and I congratulate the authors.

We sincerely appreciate the reviewer’s positive feedback. Thank you for recognizing the clarity and sufficiency of our results, methods, and statistical analysis.
