# Supplementary material for: EEG microstate transition cost correlates with task demands
Source: PLoS Comput Biol. 2024 Oct 10;20(10):e1012521. doi: 10.1371/journal.pcbi.1012521 (PMC11495555; doi:10.1371/journal.pcbi.1012521)
Supplement: Attachment — Submitted filename: Response_rewievers_second_round.pdf [file pcbi.1012521.s007.pdf]

Dear Dr. Christoph Strauch,
Dear Dr. Lyle Graham,

Thank you very much for your positive feedback concerning our manuscript. We are delighted to hear that the reviewers appreciated the improvements made during the initial revision.

We are grateful for the constructive comments provided and have carefully addressed the remaining minor points raised in this round of review. Attached, you will find our detailed responses to the reviewers' additional comments and the revised manuscript.

Sincerely,
Giacomo Barzon
Ettore Ambrosini
Antonino Vallesi
Samir Suweis

Reviewer #1:

The revised version of Barzon et al. has answered most of my questions.
I still have a few comments and minor requests.

We appreciate the reviewer's thoughtful feedback and are glad to hear that the revised version of our manuscript has addressed most of their questions.

1) Re entropy rate:
I thank the authors for their additional analyses. The finding of a maximum entropy rate during rest is quite surprising, despite the literature cited by the authors. None of the three cited papers computes the entropy rate of a neurophysiological time series (EEG or BOLD). Escrichs et al. do not use entropy at all.
The entropy rate of a microstate sequence is expected to be lower in the resting state as the EEG becomes faster during cognition, as reported in Jia et al.
Together with the unusual finding of microstate-A dominance in the resting state, I wonder if the resting state was an eyes-open resting state or had low alpha power for some other reason? I couldn't find information about eyes open/closed. Please add this.

I think the reader should be informed that the resting state in this study has these somewhat unusual features, to facilitate future comparisons. Please add this to the Discussion.

We thank the reviewer for the insightful comment regarding the resting state conditions in our study. We confirm that the resting state in our experiment was conducted with eyes open, which likely contributed to the reduction in alpha band power. We agree that this is a significant detail that should be highlighted to facilitate future comparisons. Accordingly, we have added a clarification in the Discussion section.

2) Re KL-divergence:
The additional findings presented in Fig. S3 show that KL-divergence between the microstate distributions alone seems to perform quite well as an estimator of cognitive load. Re-iterating my request from the first review, could the authors please quantify which method is more effective in measuring cognitive cost? E.g. by comparing the effect size. I think this should also be added to the Discussion where Fig. S3 is just briefly mentioned. The reader should learn that it performs with a similar efficacy (or better than?) the more advanced Schroedinger bridge method although KL-divergence ignores the transition path. The simple method performs nicely and is closely related to the authors' approach. It shows that cognitive load is already encoded in the distance between the distributions, independent of the optimum path found between them.

As suggested by the reviewer, we performed the same analyses on the Kullback-Leibler (KL) divergence instead of transition costs. However, these analyses did not reveal significant effects of congruency ($t = -1.74$, $p = .0833$, $d = -0.26$) or the congruency by PC interaction ($t = -1.33$, $p = .1841$, $d = -0.20$) on KL divergence. Additionally, the Stroop effects in KL divergence values were not significantly associated with participants' performance, as assessed by their Stroop effects in response times ($t = 0.19$, $p = .8467$, $d = 0.03$).

Based on these results, we believe that while the KL divergence provides some insights, it does not perform as effectively as the more advanced Schrödinger bridge method in measuring cognitive cost. We have added a discussion of these findings in the revised discussion.

3) Re entropy production:
Not sure if the author comment "However, to our knowledge, this measure has not yet been explored in the EEG field" was meant to say in the EEG microstate field?
Anyway, the technique has been used for non-human primate EEG data (https://doi.org/10.1093/cercor/bhac177) and has also been used to demonstrate irreversibility and non-equilibrium dynamics in EEG microstate time series in Hermann et al. (https://doi.org/10.1007/s10548-023-01023-1).
These should be cited in the Discussion.

We appreciate the reviewer's feedback and thank them for bringing these publications to our attention. We were not previously aware of these references, but we have now incorporated them into the revised version of our manuscript.

Minor:
The time axis in Fig R4 cannot be ms but thank you very much for clarifying the question.

The reviewer is correct, and we apologize for the oversight regarding the time axis in Fig R4.

Thanks for all the extra work that went into the revision. This is a very interesting approach and I'm looking forward to reading future research with it.

We thank again the reviewer for the overall feedback and for acknowledging the effort invested in the revision.

Reviewer #2:

Congratulations to the authors for the improvement of the manuscript. I am now much more convinced by the results and the readability of the paper has greatly improved. I only have a few minor points left:

We sincerely thank the reviewer for the encouraging feedback and for acknowledging the improvements in our manuscript. We greatly appreciate your thoughtful comments and are pleased that the revisions have contributed to both the clarity and the robustness of the results.

- The new sentence in the abstract could be merged with the previous one for something more concise but still informative on the nature of the task

We thank the reviewer for the suggestion. We have merged the new sentence in the abstract with the previous one to create a more concise and informative description of the task.

- The results of the mixed models in section "Microstate reconfiguration during task" are now hard to follow, maybe the authors could only report coefficients and SE and report p values and cohen's d in a table). This would make these results clearer both for those who just want a quick summary and those who want to dig into the details and can look up the table.

We thank the reviewer for your valuable suggestion. As recommended, we have revised the "Microstate reconfiguration during task" section by reporting the coefficients and standard errors in the text, while presenting the p-values and Cohen's d in Table S1. We concur that this adjustment has enhanced the readability of the section.

- Bonini, Francesca, et al. "Action monitoring and medial frontal cortex: leading role of supplementary motor area." Science 343.6173 (2014): 888-891. Might be an interesting reference for the discussion for the modification of states G and F (3rd paragraph in the discussion)

We appreciate the reviewer’s input. The reference by Bonini et al. (2014) has been incorporated into the discussion.

Reviewer #3:

I thank the authors for addressing my comments. I have no further remarks.

We sincerely thank the reviewer for the feedback. We are pleased that our responses have addressed the comments, and we appreciate the time and effort invested in evaluating our manuscript.
